# Community-Level Income Inequality and HIV Prevalence among Persons Who Inject Drugs in Thai Nguyen, Vietnam

**DOI:** 10.1371/journal.pone.0090723

**Published:** 2014-03-11

**Authors:** Travis W. Lim, Constantine Frangakis, Carl Latkin, Tran Viet Ha, Nguyen Le Minh, Carla Zelaya, Vu Minh Quan, Vivian F. Go

**Affiliations:** 1 Johns Hopkins Bloomberg School of Public Health, Department of International Health, Baltimore, Maryland, United States; 2 Johns Hopkins Bloomberg School of Public Health, Department of Biostatistics, Baltimore, Maryland, United States; 3 Johns Hopkins Bloomberg School of Public Health, Department of Health, Behavior and Society, Baltimore, Maryland, United States; 4 Johns Hopkins Bloomberg School of Public Health, Department of Epidemiology, Baltimore, Maryland, United States; 5 Thai Nguyen Center for Preventive Medicine, Thai Nguyen, Vietnam; UCL Institute of Child Health, University College London, United Kingdom

## Abstract

Socioeconomic status has a robust positive relationship with several health outcomes at the individual and population levels, but in the case of HIV prevalence, income inequality may be a better predictor than absolute level of income. Most studies showing a relationship between income inequality and HIV have used entire countries as the unit of analysis. In this study, we examine the association between income inequality at the community level and HIV prevalence in a sample of persons who inject drugs (PWID) in a concentrated epidemic setting. We recruited PWID and non-PWID community participants in Thai Nguyen, Vietnam, and administered a cross-sectional questionnaire; PWID were tested for HIV. We used ecologic regression to model HIV burden in our PWID study population on GINI indices of inequality calculated from total reported incomes of non-PWID community members in each commune. We also modeled HIV burden on interaction terms between GINI index and median commune income, and finally used a multi-level model to control for community level inequality and individual level income. HIV burden among PWID was significantly correlated with the commune GINI coefficient (r = 0.53, p = 0.002). HIV burden was also associated with GINI coefficient (β = 0.082, p = 0.008) and with median commune income (β = −0.018, p = 0.023) in ecological regression. In the multi-level model, higher GINI coefficient at the community level was associated with higher odds of individual HIV infection in PWID (OR = 1.46 per 0.01, p = 0.003) while higher personal income was associated with reduced odds of infection (OR = 0.98 per $10, p = 0.022). This study demonstrates a context where income inequality is associated with HIV prevalence at the community level in a concentrated epidemic. It further suggests that community level socioeconomic factors, both contextual and compositional, could be indirect determinants of HIV infection in PWID.

## Introduction

The ecologic relationship between health and wealth has been well characterized in the global development field [1]. The literature suggests that the cycle is self-perpetuating. Societies with higher wealth may have better living, working and housing conditions, have less stress, and are able to purchase better health care. In turn, a healthy society affords more productive workers and lower economic toll for health spending [2], [3]. The potential relationship between health and absolute wealth has been shown for various outcomes and populations [4], [5], [6]. In addition to the health and wealth gradient, the distribution of wealth within a society, may influence health. In the US, at the state level, income inequality has been shown to be associated with poorer self assessed health and higher mortality [7], [8], as well as Chlamydia and AIDS case rates [9]. Indeed, Wilkinson [10], [11], [12] has argued that relative wealth is more important for mortality and premature mortality than absolute wealth, especially in developed OECD countries.

The relationship between income and HIV is more complex than that between income and health, more generally. Some studies in generalized HIV epidemics in sub-Saharan Africa have found no association; others show that poverty is associated with lower HIV prevalence rates, while higher socioeconomic status indicators such as education and income are associated with higher HIV prevalence [13], [14].

More recently, studies have found that HIV prevalence is more strongly associated with income inequality rather than with absolute level of income [15], [16], [17], [18], [19]. Most studies on income inequality and HIV prevalence to date have focused on generalized epidemics, primarily in sub-Saharan Africa, where the unit of analysis was a country. Some subnational and multi-level analyses have studied the relationship between income inequality and HIV prevalence in Malawi, Tanzania, and Sub-Saharan Africa [20], [21], [22]. An outstanding question is whether income inequality is associated with HIV prevalence in the context of a concentrated epidemic, where the larger group contributing to the income calculation may differ substantially from the sub-group primarily contributing to the HIV prevalence calculation.

Since the first outbreak of HIV was reported in 1993 [23], the HIV epidemic in Vietnam has been concentrated among key populations at high risk, especially persons who inject drugs (PWID), who have an HIV prevalence as high as 30% according to national surveillance surveys [24]. Although Vietnam is officially a socialist state, due to its ongoing reforms under *Doi Moi* (renovation), its goal of “market-driven socialism” has driven rapid economic development that has reportedly benefitted the wealthy more than the poor [25]. Economists suggest that this transient state of rapid development can result in wealth disparities [26].

In this study, our objective is to examine the relationship between income inequality and HIV prevalence at the community level in the northern province of Thai Nguyen, and to determine the effect absolute level of income on this relationship, if any. Given results of cross-country studies [15], [16], [17], [18], [19], we hypothesize that we will also see higher HIV prevalence among PWID in communities with higher income inequality.

## Methods

This study was a secondary data analysis of the cross-sectional survey administered at the baseline visit of the Prevention With Positives Project, for a four-arm randomized community trial for HIV prevention and stigma reduction in Thai Nguyen Province, Vietnam. Between August 2009 and January 2011, we recruited 1674 male PWID using outreach staff and participant peer referral. 1349 non-PWID community members (hereafter, “community members”) were systematically sampled from the fifth through seventh households to the right of the houses of HIV-positive PWID. All participants provided written informed consent. Sample consent forms are available upon request. Sample sizes were calculated for the parent study to detect a scientifically meaningful difference in risk behaviors and stigma measures with 20% drop-out, 80% power, and a cluster-randomized design.

To be eligible for analysis, PWID had to be male, injected drugs at least once in the past six months, and be 18 years or older at enrollment. PWID were excluded if they had previous participation in an HIV prevention, substance abuse program, or peer educator intervention, or if they had cognitive or psychological impairments. Community members of both sexes were eligible if over 18 years of age.

All participants completed interviewer-administrated surveys including socioeconomic and demographic questions. Community members were asked about their average monthly incomes from all jobs and businesses, and also their incomes from supplemental sources such as government assistance and pensions; the amounts were summed to obtain total average monthly income. Interviewers were trained to probe for additional sources of income. PWID were tested for HIV using Bio-Rad rapid antibody test. Positive results were confirmed using Bio-rad ELISA according to the manufacturer's instructions at the Center for Preventive Medicine laboratory in Thai Nguyen, and post-test counseling was provided.

The study was approved by the Johns Hopkins Bloomberg School of Public Health Institutional Review Board and the Thai Nguyen Center for Preventive Medicine Institutional Review Board.

We conducted semi-structured key informant interviews with six study staff recruiters familiar with the Thai Nguyen communes, as well as provincial health officials at the Thai Nguyen Center of Preventive Medicine, to help interpret quantitative results, with an emphasis on describing communes that were inconsistent with quantitative results. Informants were asked about commune characteristics such as population density, types of employment and economic activity, level of development, presence of PWID gathering sites, and presence of HIV programs and treatment sites. Answers were cross checked against the project manager's own knowledge and inconsistencies between informants were identified and reconciled with additional field staff familiar with the area.

## Methods - Data analysis

### Variables

The GINI coefficient for income inequality was calculated for each commune from the self-reported incomes of the non-PWID community members using standard methods [27], [28], by calculating the areas bound by the Lorenz curves as a proportion of total area under the equality line. PWID are estimated to comprise less than 1% of the total population of Thai Nguyen [24], and their financial situation may not be representative of the commune as a whole, therefore, PWID participant's incomes were excluded from the calculation of the GINI coefficients.

The sample prevalence of HIV was calculated as a simple proportion of total number of PWID who tested positive in our study, divided by the total number of PWID enrolled, for each commune. We did not use random sampling, therefore our use of the term “prevalence” in our analysis in this paper refers to sample prevalence rather than true population prevalence of HIV.

To adjust for the different sample sizes of individual communes, we applied a first-order adjustment multiplier of n/(n-1) [29] to account for the possible underestimation of GINI coefficients from small sample sizes. We also applied analytic weights for the outcome of HIV prevalence, based on the number of PWID enrolled from each commune.

### Ecological analysis

Scatterplots of the communes were constructed to examine the ecologic relationship between community-level income inequality and HIV prevalence among PWID. Correlations between income inequality and HIV prevalence were calculated using weighted pair-wise Pearson's correlations.

### Regression

Ordinary least squares (OLS) linear regression of HIV prevalence on adjusted GINI coefficient was fit using STATA 11 and weighted by the number of PWID in the commune. Regression coefficients for GINI and commune income were scaled for interpretation. We created a GINI*median income interaction term by multiplying the coefficients in order to study the interaction between absolute and relative income. DFIT values were predicted for regression models to explore sensitivity to excluding outliers, but no effect on overall magnitude, direction, or statistical significance was found. The multi-level regression was fit using xtlogit with unstructured correlation in STATA 11 with random intercept for HIV prevalence among PWID in different communes. 59/1674 PWID (3.5%) had missing responses and could not be included in the regression; we deemed this proportion to be too low to affect the result.

## Results

In our study sample of 1674 PWID, HIV prevalence was 31.2%. Individual socioeconomic characteristics of PWID are shown by HIV status in Table 1. Being aged 30–39, having total monthly income <$100 USD, and less than high-school education was associated with higher Individual-level odds of being HIV positive (all p<0.01). Ever injecting with a used needle was also associated with 1.9 fold higher odds of HIV infection (p<0.001).

**Table 1:** Individual-level socioeconomic and demographic characteristics of PWID associated with HIV infection in Thai Nguyen province, Vietnam.

	Number	HIV positive	OLS Univavriate Odds ratio of HIV infection (SE)	p-value (χ^2^)
Age				
18–30	391	30.7%	Ref	
30–40	839	35.4%	1.24 (0.16)	
>40	440	23.6%	0.70 (0.11)	<0.001
Monthly Income (USD)				
<$100	970	34.4%	Ref	
$100–200	528	25.3%	0.68 (0.08)	
>$200	118	24.6%	0.62 (0.14)	0.002
Education				
Primary	169	35.5%	Ref	
Secondary	802	35.7%	1.00 (0.18)	
Graduated High school	582	25.3%	0.61 (0.11)	
College or higher	118	23.7%	0.57 (0.15)	<0.001
Employment status				
Full-time	872	29.3%	Ref	
Part-time	183	33.2%	1.20 (0.17)	
Unemployed	83	44.3%	1.92 (0.34)	
Retired, disability, student	12	14.3%	0.40 (0.31)	0.001
Using shared needle				
Never	1,117	26.5%		
Ever	554	40.6%	1.90 (0.21)	<0.001

Our study sampled from 32 total communes in Thai Nguyen province, both urban and rural. The sample HIV prevalence among PWID in the 32 communes in Thai Nguyen ranged from 4.3% to 63.6%. This range is approximately consistent with wide ranges of HIV prevalence among PWID between different provinces of Vietnam [24]. The variability in HIV prevalence between communes suggested that a commune-level ecologic analysis of HIV prevalence is feasible.

The GINI coefficient for the full sample, across communes, was 0.42 which is higher than the coefficient estimated from government sources [26]. By individual commune, the coefficients ranged from 0.19 to 0.49, and the first-order adjusted GINI coefficient ranged from 0.28 to 0.5. This variability likewise suggested that a commune-level ecologic analysis of inequality is feasible.

**Figure 1:**
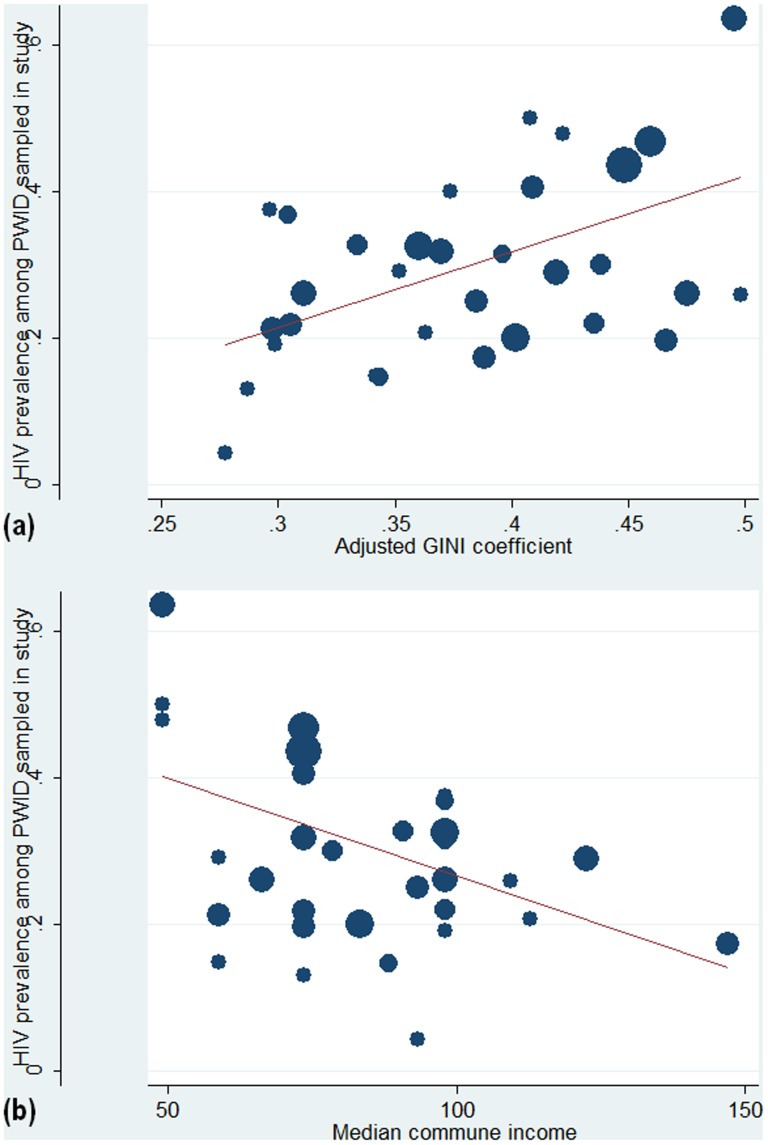
Ecological scatterplots show the relationship between HIV prevalence in our study sample of PWID, and the distribution or level of income for the 32 communes in Thai Nguyen, Vietnam. A, Crude GINI coefficient is shown along the X-axis. B, GINI coefficients adjusted with a first-order correction factor to account for variable and small sample sizes shown along the X-axis. C, median commune income is shown along the X-axis. Size of the circle represents weighting according to the total number of PWID enrolled from the commune.

To examine the relationship between income inequality and HIV prevalence at the ecologic level, we created a scatterplot of the communes and calculated the correlation coefficient (Figure 1 A–B). There is a statistically significant positive correlation of 0.53 (p = 0.002) between HIV prevalence among PWID and the income inequality level of communes, weighted by population. The positive correlation and statistical significance also holds for Spearman's rank correlation, which we used as a check for sparseness of the data (calculation not shown).

In order to assess the commune-level effect of absolute wealth on HIV prevalence, we plotted the median income to capture the former and GINI coefficient to capture the latter. Median monthly commune income, in USD, is inversely correlated with HIV prevalence (r = −0.45, p = 0.01), accounting for the sample size of each commune (Figure 1C). We chose median income as a measure of central tendency that is less affected by the distribution or skew of incomes.

To disentangle the effect of relative income distribution from the effect of absolute level of income, we used linear regression with communes as the unit of observation, to model HIV prevalence as a function of both GINI coefficient and commune median income level. We also included an indicator of rural vs. urban in the model to adjust for possible socioeconomic differences that were inherent to urbanity, such as the organization of the community. The result of the regression is shown in Table 2. An increase in first-order corrected GINI coefficient of 0.10 is independently associated with an 8.2% increase in HIV prevalence among PWID (p = 0.008), controlled for median income of the commune. An increase in $10 USD median monthly income is associated with a 1.8% decrease in HIV prevalence among PWID (p = 0.023).

**Table 2:** Linear regression of HIV prevalence on GINI coefficient, median income, and rural vs. urban for communes in Thai Nguyen, Vietnam (N = 32).

Predictor	Coefficient	p value	95% CI, lower	95% CI, upper
GINI coefficient (/10)	0.082	0.008	0.023	0.141
Median monthly income, USD (*10)	−0.018	0.023	−0.033	−0.0026
Urban (vs. rural)	−0.062	0.09	−0.151	0.361

To check for the influence of outlier communes, we predicted the DFIT values for the regression in Table 2. 29/32 communes were below the DFIT cutoff value for outlier influence. However, excluding the three influential communes from the regression in Table 2 did not influence on the overall result.

We wanted to explore possible commune characteristics that could explain these associations between median income and HIV infection, and between income inequality and HIV infection among PWID. One possibility is that the relationship is affected by a differential availability of health services [15]. Thus, we tested whether communes containing HIV services also differed in median incomes or income inequality. We found no relationship between income inequality and the presence of health services by commune. However, communes with hospitals which provided HIV-specific services had significantly higher median income (student's t-test of difference in means, average + US $27.53 higher, p = 0.004) compared to communes that did not have such services.

In order to characterize communes that did not conform to the overall positive association between income inequality and HIV infection among PWID, we conducted key informant interviews among study field staff, to explore qualitative characteristics of the 32 communes. We found that HIV infections tended to be highest in communes with a higher number of gathering spots of PWID as identified to recruiters through PWID study participants. Gathering spots are areas where PWID congregate to buy, sell or share drugs, and to inject; the presence of many of these sites may suggest larger or denser PWID networks, and may reflect increased opportunities for group injecting and needle sharing. “Exception” communes with high inequality but low HIV prevalence, or communes with low inequality but high HIV prevalence (lower right quadrant and upper left quadrant of Figure 1B, respectively) were more likely to have HIV prevalence driven by numbers of known PWID gathering sites, irrespective of their income inequality level. Moreover, the presence of PWID gathering sites often co-occur with a qualitatively described concentration of marketplaces and/or small businesses in particular communes. Lastly, communes with higher inequality and lower prevalence among PWID were characterized by key informants as having a more “scattered” population density. Other commune-level characteristics had no clear qualitatively described relationship with HIV infection or inequality.

We next fit a regression model of GINI coefficient and median commune income that included an interaction term, in order to determine whether the effect of income inequality differed by median commune income (Table 3). The interaction term between GINI coefficient and median commune income was negative and statistically significant, indicating that for communes of higher median income, the impact of income inequality on HIV prevalence is attenuated; conversely, for communes of lower median income, the impact of income inequality on HIV prevalence is exacerbated. The regression coefficient for the GINI (0.359, 95% CI 0.11–0.61) and for the linear combination of GINI and interaction term (0.324, 95% CI 0.10–0.54) were both positive and statistically significant. Furthermore, between communes with median monthly income differing by $103 or more, this negative interaction term reduces the magnitude of the GINI – HIV association to 0; in our sample, however, only the very wealthiest communes and very poorest communes reach this difference. Conversely, the regression coefficient for median commune income (0.122, 95% CI −0.002–0.246) and for the linear combination of median income and interaction term (0.087, 95% CI −0.006–0.18) are marginally statistically significant. This indicates that the previous relationship between median income and HIV in fact varies by income inequality.

**Table 3:** Linear regression of HIV prevalence on GINI coefficient, median income, GINI-median income interaction term, and rural vs. urban for communes in Thai Nguyen, Vietnam (N = 32).

Predictor	Coefficient	p value	95% CI, lower	95% CI, upper
GINI coefficient (/10)	0.359	0.006	0.110	0.607
Median monthly income, USD (*10)	0.122	0.053	−0.01	0.246
GINI-median income interaction term	−0.035	0.027	−0.066	−0.004
Urban (vs. rural)	−0.047	0.181	−1.97	0.075

Finally, to assess the relationship between income inequality and HIV infection using both individual level and community level predictors, we fit a multi-level model with communes as a level-2 cluster. Log odds of HIV infection among PWID was regressed on the statistically significant individual factors from Table 1, in addition to the statistically significant community level factors from Table 2. The results of the model are shown in Table 4. In the multi-level model, $10 higher monthly personal income of PWID was significantly associated with 0.023 lower log-odds (2.4% lower odds) of HIV infection (p = 0.009). Furthermore, when personal income was included in the model, median commune income was no longer a significant predictor, so it was removed from the model. GINI coefficient for income inequality was also a commune-level predictor of higher odds of HIV infection in PWID; an increase in GINI coefficient of 0.1 was associated with a 0.39 higher log-odds (46% higher odds) of HIV infection among PWID (p = 0.002).

**Table 4:** Multi-level logistic model that regresses odds of HIV infection among PWID on significant individual-level and community-level predictors in the same model.

	Predictor	Coefficient	p value	95% CI, lower	95% CI, upper
Individual level	Monthly personal income, USD (*10)	−0.020	0.022	−0.037	0.003
	Employment status (vs full-time)				
	Part-time	0.169	0.277	−0.136	0.474
	**Unemployed**	0.821	0.000	0.445	1.197
	Unable to work/disability/student	−0.776	0.322	−2.309	0.758
	Highest Level of School (vs primary)				
	School: Secondary	0.095	0.614	−0.276	0.467
	School: High school	−0.367	0.071	−0.765	0.031
	School: College or higher	−0.419	0.150	−0.991	0.152
	Age	−0.012	0.134	−0.027	0.004
	**Ever used a used needle**	0.493	0.000	0.260	0.727
Commune level	Median commune income	−0.006	0.094	−0.014	0.001
	**Adjusted GINI coefficient (/10)**	3.804	0.003	1.328	6.280
	Urban (vs. rural)	−0.215	0.187	−0.534	0.104
Random effect of commune					
	Model paramater	Variance	Var Std Err.	95% CI, lower	95% CI, upper
	Random commune intercept	0.069	0.043	0.020	0.234
	Likelihood ratio test: Probability that the random commune intercept model has lower log-likelihood than regular logistic regression, p = 0.0061				

(N = 1615 PWID clustered in 32 communes.).

## Discussion

In this paper, we present one of the few analyses demonstrating the association between income inequality and HIV prevalence at a community level of aggregation, where the social determinants of health are primarily believed to operate [30]. The positive relationship between HIV prevalence and income inequality has been shown in a number of other studies in which the country was the unit of aggregation [13], [14], [16], [18], [19], [31]. In those studies, similar to our results, income inequality was also associated with HIV prevalence, and was not associated with absolute income on a per-capita (mean) basis. However, we also found HIV infection was associated with median community income and that effect appears to be confounded by the presence of hospitals providing HIV services within wealthier communes.

Furthermore, past studies have often focused on generalized epidemic settings, where the income data used in the calculations come from the same population providing the HIV prevalence data. In our analysis, we used the setting of Vietnam to study an epidemic concentrated primarily in PWID; therefore we believe that income inequality is more likely acting as a contextual socioeconomic effect in the community as a whole, not just a compositional effect of individual absolute incomes of PWID.

The strength of the relationship between HIV prevalence and income inequality was statistically significant and robust to several different specifications such as first-order adjustment of the GINI coefficient, weighted or unweighted by commune sample size, and Spearman's vs. Pearson's correlations. Notably, the inequality-HIV relationship persisted when controlling for median absolute income level in a regression model. Conversely, higher community median income was significantly associated with lower HIV prevalence among PWID, controlling for GINI coefficient. These results suggest that both general economic development, if it increases overall wealth, and reduction of economic inequalities might indirectly reduce HIV infection independently of one another.

While this study does not have data available to determine the mechanism through which income inequality is associated with higher HIV infection, there are a number of explanations to consider. One possible explanation may be that higher income inequality leads to a break-down of social norms, which in turn leads to greater social problems generally, including drug use. Another mechanism may be a community level characteristic proposed previously [15]. For example, higher inequality in a community could coincide with weakness in its social safety net, welfare payments, and quality of public resources spent on health, in turn resulting in more and/or poorly managed HIV infections [7], [32]. In Vietnam it seems unlikely that standardized government health care clinics would vary much between communes; however, there are still opportunity costs, such as missing work, to attending care even if user fees are abolished. Another explanation is that communities with higher income inequality have lower social capital and lower mutual trust, which in turn might decrease the diffusion of personal risk across society through reduced knowledge transfer, reduced cooperation and support, and reduced enforcement of health norms [33]. In this case, instead of applying a direct economic intervention, targeted programs to enhance social capital [34], or community participatory programs that include social capital components [35], [36] could be introduced. However, more research is required to understand the relationship between social capital and income inequality.

Past ecological studies found that income inequality, but often not absolute level of income, is associated with HIV prevalence at the country level [13], [16], [18], [19]. By fitting an interaction term between income inequality and absolute income, we demonstrated that relative and absolute income interact at the community level. Therefore, the relationship between inequality and HIV prevalence is attenuated for communities with a higher overall income, and exacerbated for communities with a lower overall income. One possible explanation is that in higher-income communes, there is sufficient income for everyone to meet basic health and welfare needs, even if not distributed equally; whereas in low-income communes, unequal distribution of incomes leaves the poorest below some threshold of meeting minimum health and welfare needs. Another explanation may be that the confluence of inequality and poverty create a situation in which low overall resources, and lower propensity to redistribute those resources, force PWID to adopt different types of injecting networks to pool resources, that are more concentrated and interconnected, resulting in higher needle sharing and other risk behaviors, and higher HIV transmission rates.

The potential role of networks is underscored by our qualitative findings. Qualitatively, we found that communes with low income inequality could have high HIV infection among PWID if the commune had popular PWID gathering spots. The injecting risks and drug use behaviors facilitated by these gathering spots may modify the relationship between income inequality and HIV infection in PWID. Additionally, the few exception communes with high inequality but low HIV infection in PWID were described as sparsely populated, which could mean that PWID in general are more dispersed in the area and therefore had lower density networks, a factor in HIV infection [37], [38], [39]. However, we did not systematically quantify or survey PWID gathering spots or PWID networks in this study, so additional research elucidating how inequalities affect PWID network structures is warranted.

The results of the multi-level model suggest that commune-level income is less important to a PWID for HIV infection than his own personal income. Both commune-level income inequality and personal income are statistically significant in the multi-level regression model suggesting that they each operate independently from one another. In other words, PWID who live in communes with a high level of income inequality will have higher odds of HIV infection regardless of their level of personal wealth. This finding is consistent with multi-level analyses of inequality and HIV infection in generalized epidemics in Sub-Saharan Africa [21] and Malawi [20], in which the association between HIV infection and inequality persists when accounting for individual-level economic factors.

Limitations to our study should be noted. Correlation does not imply causation, and like other social determinants of health, income inequality at any level of aggregation must necessarily operate through sociologic mechanisms that are still not well understood. Another limitation is the potential measurement error in “income.” While we took efforts to simplify the income questions on our survey and trained our interviewers to verify truthful income reports and probe about various sources of income, it is possible that participants misrepresented their income. However, if they did so, we believe that such misrepresentation occurred in a uniform direction that may have shifted the mean, but not the distribution, of incomes in our survey. Finally, because we chose to study income inequality at the community level within one country, our study results cannot necessarily be generalized outside of Vietnam.

Despite these limitations, our study also had several strengths. First, through the recruitment design, community members were selected for the physical proximity of their household to the household of the PWID. The community sample may be a more accurate reflection of participants' microenvironment than a study of two people living on opposite sides of their communes who do not interact. Secondly, we have disassociated the effect of individual-level income of PWID on HIV prevalence by calculating our GINI coefficients using the incomes of the non-PWID community members. This helps protect against possible reverse causality; although HIV in PWID could widen the gap between PWID income and community income, it is unlikely to widen the gap between incomes of non-PWID community members. Thirdly, by choosing to utilize community-level data rather than of country-level data, we have eliminated many cultural and social factors that may be confounders of this relationship. For example, a cultural norm that would increase inequality and also increase HIV infections is expected to be fairly homogenous within this region but could differ between countries.

There is mounting evidence from psychology, sociology, and public health supporting a mechanism in which wealth and wealth disparities in a community could potentially decrease social capital [33], [40] and community trust [12], and increase stigma [41]. Elucidation of how these mechanisms could affect HIV infection should be pursued. There are also other types of hidden populations at higher risk of HIV infection in concentrated epidemics, such as men who have sex with men and commercial sex workers. The role that economic inequality plays in these epidemics should also be examined.

## References

[1] Bloom DE, Canning D (2000) The Health and Wealth of Nations. Science 287: 1207–1209. 10712155 10.1126/science.287.5456.1207

[2] Ruger JP, Jamison DT, Bloom DE, Canning D (2011) Health and the Economy. In: Merson MH, Black RE, Mills AJ, editors. Global Health: Diseases, Systems, Programs and Policies. 3rd ed: Jones & Bartlett. pp. 757.

[3] World Health Organization (2001) Report of the Commission on Macroeconomics and Health. Commission on Macroeconomics and Health. 213 p.

[4] Marmot MG, Smith GD, Stansfeld S, Patel C, North F, et al (1991) Health inequalities among British civil servants: the Whitehall II study. Lancet 337: 1387–1393. 1674771 10.1016/0140-6736(91)93068-k

[5] Pappas G, Queen S, Hadden W, Fisher G (1993) The increasing disparity in mortality between socioeconomic groups in the United States, 1960 and 1986. N Engl J Med 329: 103–109. 8510686 10.1056/NEJM199307083290207

[6] Singh GK, Siahpush M (2006) Widening socioeconomic inequalities in US life expectancy, 1980–2000. Int J Epidemiol 35: 969–979. 16684899 10.1093/ije/dyl083

[7] Kawachi I, Kennedy BP, Lochner K, Prothrow-Stith D (1997) Social capital, income inequality, and mortality. Am J Public Health 87: 1491–1498. 9314802 10.2105/ajph.87.9.1491 PMC1380975

[8] Kennedy BP, Kawachi I, Prothrow-Stith D (1996) Income distribution and mortality: cross sectional ecological study of the Robin Hood index in the United States. BMJ 312: 1004–1007. 8616345 10.1136/bmj.312.7037.1004 PMC2350807

[9] Holtgrave DR, Crosby RA (2003) Social capital, poverty, and income inequality as predictors of gonorrhoea, syphilis, chlamydia and AIDS case rates in the United States. Sex Transm Infect 79: 62–64. 12576618 10.1136/sti.79.1.62 PMC1744600

[10] Lobmayer P, Wilkinson R (2000) Income, inequality and mortality in 14 developed countries. Sociology of Health & Illness 22: 401–414.

[11] Wilkinson RG (1997) Socioeconomic determinants of health. Health inequalities: relative or absolute material standards? BMJ 314: 591–595. 9055723 10.1136/bmj.314.7080.591 PMC2126067

[12] Wilkinson RG, Pickett KE (2007) The problems of relative deprivation: why some societies do better than others. Soc Sci Med 65: 1965–1978. 17618718 10.1016/j.socscimed.2007.05.041

[13] Fox AM (2010) The social determinants of HIV serostatus in sub-Saharan Africa: an inverse relationship between poverty and HIV? Public Health Rep 125 (Suppl 4) 16–24. 10.1177/00333549101250S405 PMC2882971 20629252

[14] Gillespie S, Kadiyala S, Greener R (2007) Is poverty or wealth driving HIV transmission? AIDS 21 (Suppl 7) S5–S16. 10.1097/01.aids.0000300531.74730.72 18040165

[15] Holmqvist G (2009) HIV and Income Inequality: If There is a Link, What Does it Tell Us? Stockholm: Institute for Futures Studies International Policy Centre for Inclusive Growth.

[16] Nepal B (2007) Prosperity, equity, good governance and good health: focus on HIV/AIDS pandemic and its feminization. World Health Popul 9: 73–80. 18272944 10.12927/whp.2007.19293

[17] Piot P, Greener R, Russell S (2007) Squaring the circle: AIDS, poverty, and human development. PLoS Med 4: 1571–1575. 17958469 10.1371/journal.pmed.0040314 PMC2039763

[18] Sawers L, Stillwaggon E, Hertz T (2008) Cofactor infections and HIV epidemics in developing countries: implications for treatment. AIDS Care 20: 488–494. 18449828 10.1080/09540120701868311

[19] Deuchert E, Brody S (2007) Lack of autodisable syringe use and health care indicators are associated with high HIV prevalence: an international ecologic analysis. Ann Epidemiol 17: 199–207. 17174567 10.1016/j.annepidem.2006.09.005

[20] Durevall D, Lindskog A (2012) Economic Inequality and HIV in Malawi. World Development 40: 1435–1451.

[21] Fox AM (2012) The HIV-poverty thesis re-examined: poverty, wealth or inequality as a social determinant of HIV infection in sub-Saharan Africa? Journal of Biosocial Science 44: 459–480. 22273351 10.1017/S0021932011000745

[22] Msisha WM, Kapiga SH, Earls FJ, Subramanian SV (2008) Place matters: multilevel investigation of HIV distribution in Tanzania. AIDS 22: 741–748 710.1097/QAD.1090b1013e3282f3947f. 18356604 10.1097/QAD.0b013e3282f3947f PMC2789284

[23] Hien NT, Long NT, Huan TQ (2004) HIV/AIDS Epidemics in Vietnam: Evolution and Responses. AIDS Education and Prevention 16: 137–154. 10.1521/aeap.16.3.5.137.35527 15262572

[24] National Institute of Hygiene and Epidemiology, Family Health International (2007) HIV/STDs Behavioral and Biological Surveillance, 2005–2006. Hanoi: Ministry of Health.

[25] Mishra D, Đinh VT, Rab H, Đoàn VQTQH, Quillin B (2012) Taking Stock: an Update on Vietnam's Recent Economic Development. Washington, D.C.: The World Bank. 18 p.

[26] Cuong NV, Truong TN, Weide Rvd (2010) Poverty and Inequality Maps for Rural Vietnam: An Application of Small Area Estimation. Washington, D.C.: The World Bank, Development Research Group - Poverty and Inequality Team.

[27] Haughton J, Khandker SR (2009) Handbook on Poverty and Inequality. Washington, D.C.: The World Bank. 101 p.

[28] Pan American Health Organization (2001) Measuring Health Inequalities: Gini Coefficient and Concentration Index. Epidemiological Bulletin. Washington, D.C. 11370649

[29] Deltas G (2003) The small sample size bias of the GINI coefficient: results and implications for empirical research. Review of Economics and Statistics 85: 226–234.

[30] Commission on the Social Determinants of Health (2006) Closing the gap in a generation: Health equity through action on the social determinants of health. Final Report of the Commission on Social Determinants of Health. Geneva: World Health Organization.

[31] Talbott JR (2007) Size matters: the number of prostitutes and the global HIV/AIDS pandemic. PLoS One 2: e543. 17579715 10.1371/journal.pone.0000543 PMC1891093

[32] Bartley M, Blane D, Montgomery S (1997) Health and the life course: why safety nets matter. BMJ 314: 1194–1196. 9146402 10.1136/bmj.314.7088.1194 PMC2126511

[33] Barnett T, Whiteside A (2006) AIDS in the Twenty-First Century, Fully Revised and Updated Edition: Disease and Globalization: Palgrave Macmillan.

[34] Pronyk PM, Harpham T, Morison LA, Hargreaves JR, Kim JC, et al (2008) Is social capital associated with HIV risk in rural South Africa? Social Science & Medicine 66: 1999–2010. 18299168 10.1016/j.socscimed.2008.01.023

[35] Farquhar SA, Michael YL, Wiggins N (2005) Building on Leadership and Social Capital to Create Change in 2 Urban Communities. American Journal of Public Health 95: 596–601. 15798115 10.2105/AJPH.2004.048280 PMC1449226

[36] Israel B, Krieger J, Vlahov D, Ciske S, Foley M, et al (2006) Challenges and Facilitating Factors in Sustaining Community-Based Participatory Research Partnerships: Lessons Learned from the Detroit, New York City and Seattle Urban Research Centers. Journal of Urban Health 83: 1022–1040. 17139552 10.1007/s11524-006-9110-1 PMC3261295

[37] De P, Cox J, Boivin J-F, Platt RW, Jolly AM (2007) The importance of social networks in their association to drug equipment sharing among injection drug users: a review. Addiction 102: 1730–1739. 17935581 10.1111/j.1360-0443.2007.01936.x

[38] Latkin C, Mandell W, Oziemkowska M, Celentano D, Vlahov D, et al (1995) Using social network analysis to study patterns of drug use among urban drug users at high risk for HIV/AIDS. Drug and Alcohol Dependence 38: 1–9. 7648991 10.1016/0376-8716(94)01082-v

[39] Pilon R, Leonard L, Kim J, Vallee D, De Rubeis E, et al (2011) Transmission patterns of HIV and hepatitis C virus among networks of people who inject drugs. PLoS One 6: e22245. 21799802 10.1371/journal.pone.0022245 PMC3140499

[40] Reidpath DD, Chan KY, Gifford SM, Allotey P (2005) ‘He hath the French pox’: stigma, social value and social exclusion. Sociol Health Illn 27: 468–489. 15998347 10.1111/j.1467-9566.2005.00452.x

[41] Link BG, Phelan JC (2001) Conceptualizing Stigma. Annual Review of Sociology 27: 363–385.

